# Detection of circulating tumor DNA in patients with osteosarcoma

**DOI:** 10.18632/oncotarget.24268

**Published:** 2018-01-18

**Authors:** David M. Barris, Shoshana B. Weiner, Robert A. Dubin, Michael Fremed, Xusheng Zhang, Sajida Piperdi, Wendong Zhang, Shahina Maqbool, Jonathan Gill, Michael Roth, Bang Hoang, David Geller, Richard Gorlick, Daniel A. Weiser

**Affiliations:** ^1^ Department of Genetics and Department of Pediatrics, Albert Einstein College of Medicine, Bronx, NY, USA; ^2^ Computational Genomics Core, Albert Einstein College of Medicine, Bronx, NY, USA; ^3^ Department of Pediatrics, Montefiore Medical Center, Bronx, NY, USA; ^4^ Division of Pediatrics, University of Texas MD Anderson Cancer Center, Houston, Texas, USA; ^5^ Department of Orthopedic Surgery, Montefiore Medical Center, Bronx, NY, USA; ^6^ Division of Hematology/Oncology, Children's Hospital at Montefiore, Bronx, NY, USA; ^7^ Departments of Pediatrics and Genetics, Albert Einstein College of Medicine, Bronx, NY, USA

**Keywords:** circulating tumor DNA, osteosarcoma, targeted sequencing, Next Generation Sequencing, targeted therapy

## Abstract

Identification and quantification of somatic alterations in plasma-derived, circulating tumor DNA (ctDNA) is gaining traction as a non-invasive and cost effective method of disease monitoring in cancer patients, particularly to evaluate response to treatment and monitor for disease recurrence. To our knowledge, genetic analysis of ctDNA in osteosarcoma has not yet been studied. To determine whether somatic alterations can be detected in ctDNA and perhaps applied to patient management in this disease, we collected germline, tumor, and serial plasma samples from pediatric, adolescent, and young adult patients with osteosarcoma and used targeted Next Generation Sequencing (NGS) to identify somatic single nucleotide variants (SNV), insertions and deletions (INDELS), and structural variants (SV) in 7 genes commonly mutated in osteosarcoma. We demonstrate that patient-specific somatic alterations identified through comparison of tumor-germline pairs can be detected and quantified in cell-free DNA of osteosarcoma patients.

## INTRODUCTION

Osteosarcoma is the most common malignant primary bone tumor. Each year 800 new cases of osteosarcoma are diagnosed in adults and children in the United States [1]. The presentation of osteosarcoma follows a bimodal distribution, with an initial peak between ages 10 and 14 years and a second peak among individuals greater than 60 years old [2]. Since 1970, the use the chemotherapy has improved long-term survival rates from less than 20% to 70%. However, improvement in survival rates has since stagnated [3]. Prognosis of osteosarcoma is highly dependent on stage at presentation. Patients with localized disease can expect 5-year survival rates as high as 60–78%, but survival drops to 20–30% for those with metastatic disease [4]. At primary diagnosis, a very high fraction of osteosarcoma patients have malignant cells in bone marrow, with a correlation between the presence of tumor cells, clinical stage, and disease progression [5]. Metastatic disease at the time of presentation decreases long-term outcomes from 70% to less than 20% [6] underscoring the need for a comprehensive clinical assay for detection of both macro- and micro-metastases.

As with most solid tumors, the gold standard for genetic assessment of osteosarcoma is tissue biopsy [7]. However, tissue biopsy has inherent limitations. As a time intensive procedure, tissue sampling is costly, invasive, and thus limited in frequency performed. The most common site of osteosarcoma is in the extremities where risks of biopsy include bleeding, bruising, discomfort, infection, and less commonly bone fracture. Biopsies of metastatic sites, most commonly the lungs, present higher risk to the patient, with one study showing an adverse event rate of up to 17.1% for thoracic biopsies [8]. Furthermore, the mutations detected in metastatic clones can be different from the primary tumor and from other metastases [9]. Therefore, the genetic landscape discovered from tumor biopsy may not correspond to the entire tumoral cell population during prognostic assessment and longitudinal care.

As a supplement to local tissue biopsies, ctDNA isolated from blood plasma may have utility as a “liquid biopsy” of tumor burden. CtDNA is released into the bloodstream through apoptosis or necrosis of circulating tumor cells, primary tumor or metastatic lesions [10]. Anywhere from 0.01%-90% of the total cell free DNA in the bloodstream may be constituted by ctDNA [11]. In contrast to tumor biopsy, sequencing ctDNA from peripheral blood is minimally invasive and cost effective with little added risk to the patient. Because ctDNA has a short half-life, it can be used to detect changes on the scale of hours, providing real time assessment of tumor burden. Using pooled DNA from the entire tumor may also allow for assessment of tumor heterogeneity, encompassing the full spectrum of mutations in the tumor cell population [12, 13].

Furthering the potential for utility of ctDNA, many studies have correlated the quantity of ctDNA in patients’ blood with clinical outcomes [14–16]. In non-small cell lung cancer, Douillard *et al.* showed 94% concordance between ctDNA and tumor EGFR mutation status, allowing for diagnosis and targeted treatment of EGFR mutations in patients with insufficient tissue quantity [17]. Unlike most pediatric cancers that have a somatic mutation rate of 0.1 mutations/megabase, osteosarcoma has a median rate of 1.2 mutations/megabase [18]. The complex mutation landscape unique to osteosarcoma is generated by a mechanism called chromothripsis by which many somatic point mutations and structural variations are acquired in a single catastrophic event [19]. Chromothripsis is often accompanied by a pattern called kataegeis whereby multiple base mutations occur in nearby rearrangement breakpoints [20]. While ctDNA has been identified across numerous cancer types, it has not yet been studied in a cancer with such a broad mutation landscape as osteosarcoma.

Due to potentially small amounts of ctDNA in peripheral blood many ultra sensitive methods have been developed for its detection. These methods include: real-time PCR, digital droplet PCR (ddPCR), Next-Generation Sequencing (NGS) and Beads Emulsion Amplification and Magnetics (BEAMing) [15]. NGS based analysis allows for multiple somatic mutations to be identified simultaneously, allowing for a broader depiction of the tumor mutation spectrum than more targeted methods. NGS also allows for detection of copy number alterations and large rearrangements.

In this study we utilized a targeted NGS approach to detect mutations in frequently mutated genes in osteosarcoma. Chen *et al.* performed whole exome sequencing on osteosarcoma tumor samples and discovered alterations in *TP53* (95%) as well as *RB1*, *ATRX*, and *DLG2* (29–53%) [21]. Our sequencing included the introns and exons of *TP53*, *RB1*, *ATRX*, *DLG2, PTEN, MET,* and *SLC19A1*. We first compared the germline DNA (gDNA) with tissue biopsies to identify tumor-specific mutations. Using these mutations as search terms for plasma sample sequencing, we identified and quantified ctDNA in osteosarcoma patients at various time points of their treatment. This study is the first, to the best of our knowledge, to confirm the presence of circulating tumor DNA in osteosarcoma.

## RESULTS

### Patient characteristics

Cell free DNA was collected from the plasma of 10 patients with osteosarcoma (Table 1). Tumor DNA was collected from 8 of these patients. Collection from 1 patient yielded insufficient quantities of tumor DNA, resulting in poor library construction and extremely low (2x) depth of coverage and could not be further analyzed. Thus, 7 germline/tumor pairs were subjected to additional analysis for ctDNA tumor burden. The age of participating patients at time of enrollment ranged between 9–35 years. The primary site of disease varied, with 40% in the femur. 3/7 (42.9%) patients with matched tumor-germline-plasma samples, had clinical recurrence consisting of disease spread to the lungs.

**Table 1:** Patient characteristics

**A. Patients with matched tumor-germline-plasma samples**					
**Pt**	**Age (yrs)**	**Primary site of disease**	**Metastatic sites**	**Treatment protocol**	**Currently disease free (months of disease-free follow up)**
A	35	R. Forearm	None	MAP	Yes (22)
B	9	Occipital Skull	None	MAP	Yes (22)
C	18	L fibula	None	MAP	Yes (41)
D	20	L Rib	Single Lung Nodule	MAP	Yes (25)
E	20	L Femur	> 15 Lung Nodules	MAP, Dinutuximab	No, Deceased
F	10	R Femur	> 15 Lung Nodules	MAP	No
G	22	L Distal Femur	Single Lung Nodule	MAP	No
**B. Patients with matched germline-plasma samples**					
**Pt**	**Age (yrs)**	**Primary site of disease**	**Metastatic sites**	**Treatment protocol**	**Currently disease free (months of disease-free follow up)**
H	10	Multifocal	5 Lung Nodules	MAP	No, Deceased
I	23	Sacrum	None	MAP	Yes (24)
J	11	R Femur	None	MAP	Yes (10)

Note: MAP therapy consists of Methotrexate, Doxorubicin, & Cisplatin

### Sample collection

**Figure 1:**
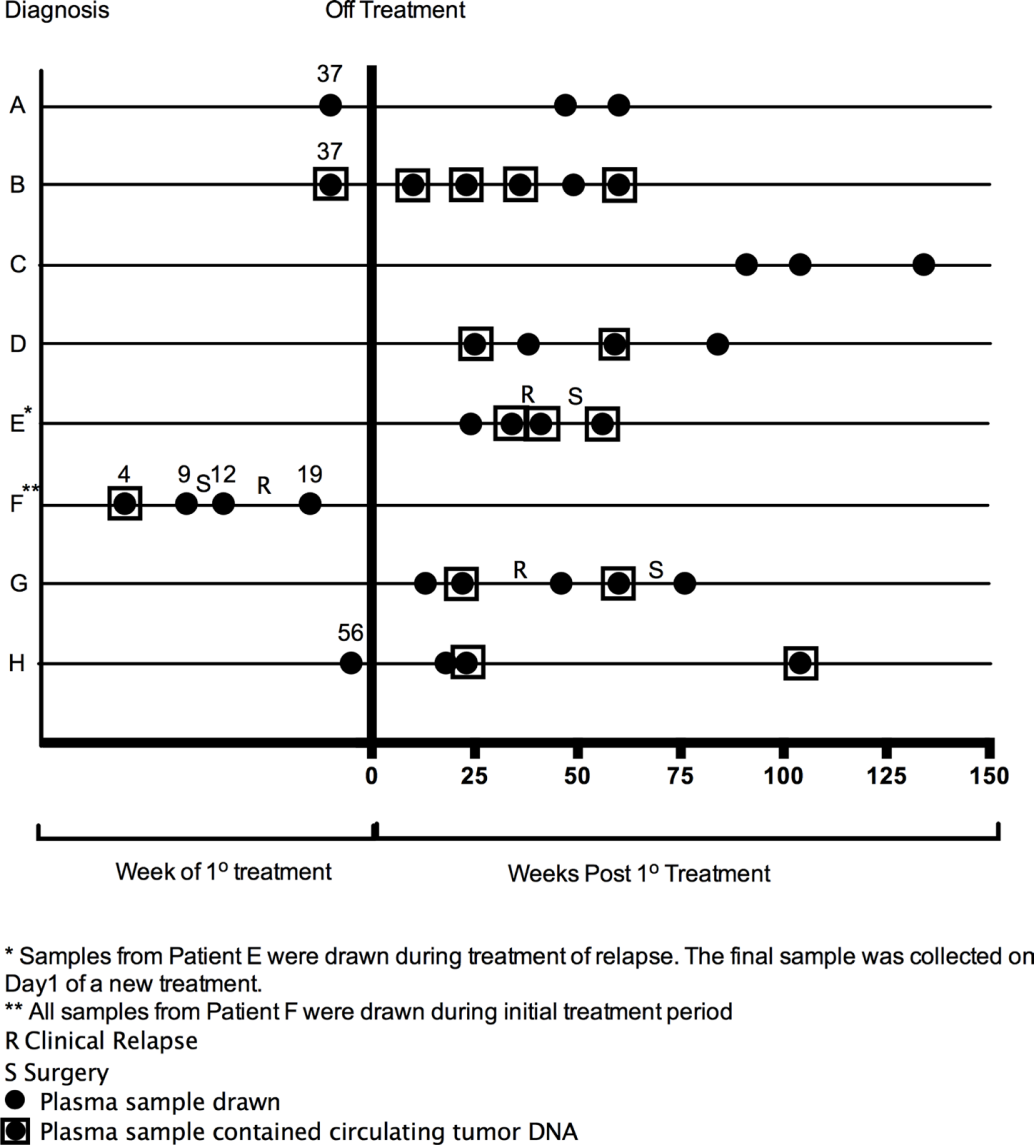
Clinical time points of plasma samples that underwent genomic analysis

Plasma samples were drawn at varying clinical time points for each patient, ranging from week 4 of treatment of primary tumor to 134 weeks post completion of treatment, as well as during treatment of recurrent disease, in the case of patient E. CtDNA was identified in 13/28 (46.4%) of samples. The relationship of ctDNA detection with clinical time course is described in Figure 1.

### Sequencing analysis

The mean depth of coverage on all samples was 698.4.1x (range 69–1,524) with a median of 635.8x (Supplementary Table 1). In patients A–G, a total of 1,331 SNVs and INDELs were discovered in the tumor DNA that were absent in the germline DNA.

A number of patient-specific somatic alterations, initially discovered through analysis of tumor-germline paired samples, were observed in ctDNA of 3 patients (E–G), generally during periods of clinical relapse. Such mutations included a translocation involving intron 1 of *TP53* and SNVs in *ATRX*, *DLG2*, and *MET*. SNVs in *DLG2* were also discovered in the ctDNA of patients B and D (Supplementary Table 2).

**Figure 2:**
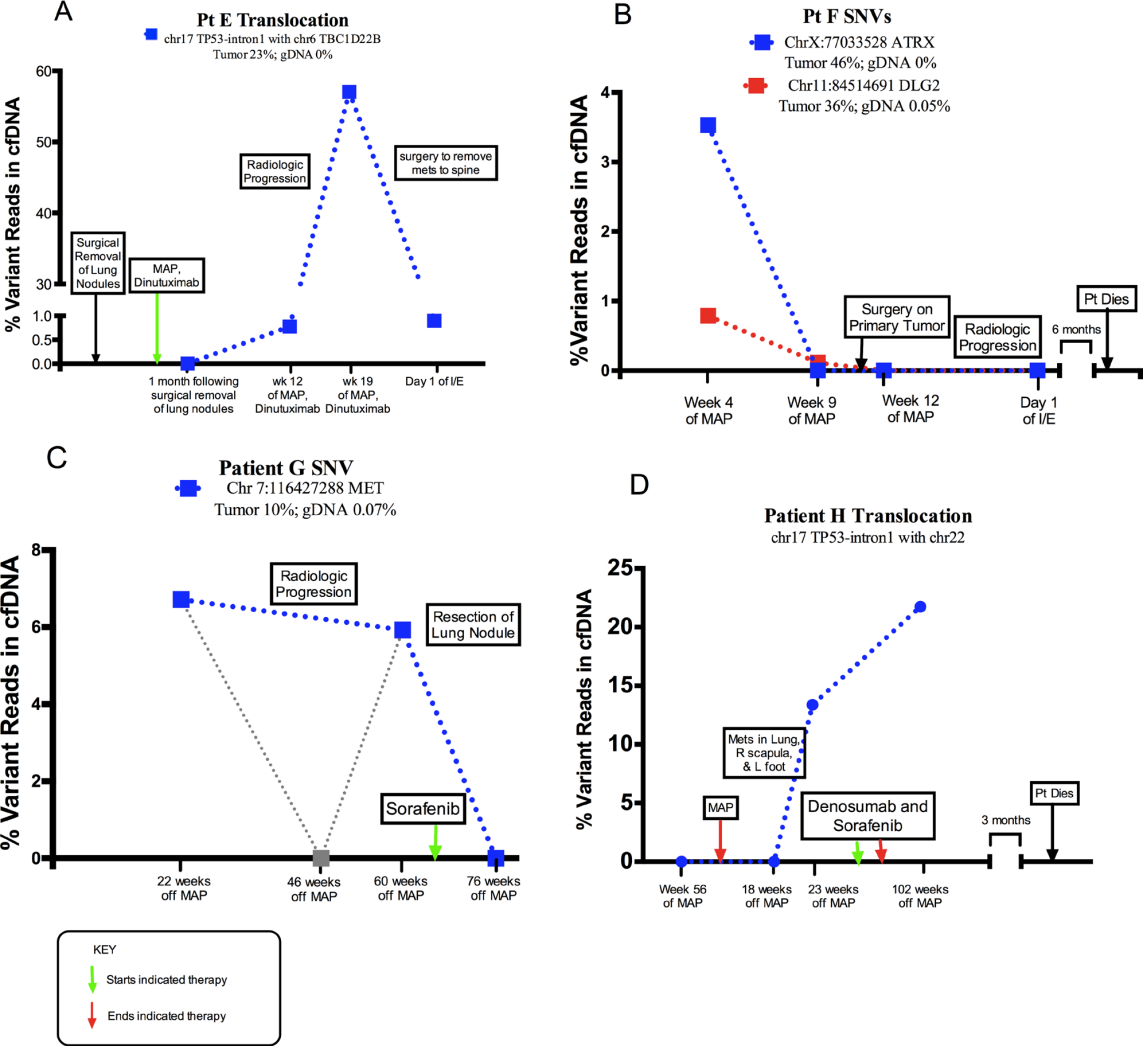
Abundance of structural alteration with associated clinical course Each sample collection is represented by large square. Dotted lines serve to visually connect time points rather than represent individual collections. (**A**) The relative quantity of a translocation (TP53) in serially collected plasma samples. The first sample was drawn following the removal of lung nodules and initiation of treatment. Over the course of the study, the patient was diagnosed with disease relapse to the spine, and underwent surgical removal of the spine metastases. (**B**) The relative quantity of two SNVs, (chr X position 77033528 ATRX gene and chr 11 position 84514691 in DLG2 gene) in serially collected plasma samples. Samples were drawn during treatment of clinically progressive disease, after surgery on the primary tumor, and following discovery of metastases to the lung. (**C**) The relative quantity of an SNV, (chr 7 position 116427288 in MET gene) in serially collected plasma samples. Samples were drawn after completion of treatment of primary tumor as well as before and after surgical removal of a lung nodule. (**D**) The relative quantity of a TP53 intron 1 translocation in serially collected plasma samples. The translocation was discovered without matched tumor DNA. Samples were drawn after completion of primary treatment and during clinical progression of disease.

Patient E tumor material contained a translocation in the first intron of *TP53* at chromosome 17 position 7583675 with chromosome 6 position 37227977 (Tumor 23%; gDNA 0%). CtDNA from patient E contained the same translocation breakpoint within intron 1 of *TP53*. The translocation was detected in 57.04% of reads in the region of the break site in one ctDNA sample obtained from this patient during a period of relapse (Figure 2A). CtDNA from Patient F contained one SNV in the *ATRX* gene (3.53% of reads) and one SNV in the *DLG2* gene (0.79% of reads) that matched tumor specific aberrations. These mutations were discovered during clinically progressive disease and became undetectable over the course of treatment (Figure 2B). Two samples of ctdDNA from patient G contained an SNV in the *MET* gene (6.72% and 5.93% of reads, respectively). These samples were collected before and after radiologic progression of disease and became undetectable after surgical removal of a lung nodule (Figure 2C). Patient G tumor sample also contained an intragenic deletion in *ATRX* spanning intron 2 through intron 15 (tumor 14%, gDNA 0%) that was not detected in circulation.

Following annotation of somatic SNVs and INDELS, pathogenicity of tumor variants was evaluated using the NCBI Single Nucleotide Polymorphism/ClinVar database (Supplementary Table 3).

### Aberrations discovered in plasma samples without using correlated DNA from a matched tumor sample

Each plasma sample was independently analyzed through Delly to identify translocations detectible without correlated tumor DNA: patient E's somatic *TP53* intron 1 translocation was confidently detected, and a new translocation was discovered in patient H, which also involved the intron 1 of *TP53* at chromosome 17 position 7588196 with chromosome 22 position 43579550 (Figure 2D). In this case, two cell-free plasma DNA samples from patient H contained this different TP53 intron 1 breakpoint during clinical relapse, yet it was absent from patient H's germline DNA and it was also absent from the patient's cell-free plasma DNA prior to relapse.

### Statistical analysis

CtDNA kinetics from the entire cohort were analyzed for summary statistics. In cases with no evidence of disease presence, 7/16 (44%) of plasma samples had detectable ctDNA. In cases of active disease, 8/16 (50%) of samples had detectable ctDNA. 2/2 (100%) of our patients with relapse during the study period had detectable ctDNA as a possible sign of relapse. In these cases, ctDNA was detected 2 months and 8 months prior to clinical detection (Figure 2A and 2C). The mean lead-time of ctDNA detection to relapse was 5 months. 2 patients in cohort had clinically progressive disease from the time of enrollment in the study through death. Both of these patients (100%) had detectible ctDNA levels in their diseased states (Figure 2B and 2D).

## DISCUSSION

NGS was performed on serially collected plasma samples with matched primary tumor material and peripheral mononuclear cells from 7 patients with osteosarcoma who were at various stages of treatment or post treatment. Each sample was analyzed for single nucleotide variants, INDELs, and larger structural variants in seven genes that are commonly mutated in osteosarcoma. 3 of the patients with germline-tumor pairs had clinical relapse during the study. We detected SNVs in the plasma of 2 relapsed patients that were present in the tumor DNA and absent in the genomic DNA. We similarly found a translocation in the plasma of the third relapsed patient. We further noted that p53 intron 1 translocations could be discovered without reference tumor DNA. Our results indicate that the targeted NGS method is capable of detecting somatic mutations and genetic aberrations in primary tissue samples and cell-free DNA samples isolated from patient plasma.

Jamal-Hanjani *et al.* used Illumina HiSeq to perform multiregion whole-exome sequencing on multiple regions of NSCLC tumors and observed extensive intratumor heterogeneity [22]. Osteosarcoma is also notable for its wide array of genetic mutations and complex tumor heterogeneity [20]. Using our targeting sequencing approach, we were able to detect pathogenic mutations in tumor material. One of the benefits of using ctDNA as a liquid biopsy is its ability to capture full tumor heterogeneity and track genomic evolution [23]. However, our study found only one to two mutations in each patient with clinical relapse. By selecting mutations in primary tumor as our search terms for ctDNA, we likely excluded mutations representative of clonal expansion and full tumor heterogeneity [24]. Matching the ctDNA to the tumor was essential to our primary endpoint of proving that NGS can identify SNVs in the plasma of osteosarcoma patients. Further study may explore detection of these mutations without limiting the search to matched mutations in the primary tumor. In addition, for plasma samples drawn during chemotherapy treatment, the portion of ctDNA in the blood stream may be diluted by the increase in cell free DNA from the necrosis and apoptosis or normal cells. This impacts the ability for NGS to call variants attributable to ctDNA. In order to validate the use of ctDNA as a prognostic marker additional baseline samples from treatment naïve patients will be crucial.

We believe the SNVs that were discovered in the plasma of patients B and D despite lack of clinical relapse may be explained by an intact host immune response to extremely low levels of circulating tumor cells. In a mouse model of osteosarcoma, those with SCID had a higher rate of metastasis, indicating an important role for T cell based immune surveillance in preventing metastases [25]. Indeed, early lymphocyte recovery represents a significant prognostic indicator for osteosarcoma [26]. We note that patients B and D both possess a higher risk of relapse independent of their circulating tumor DNA. The unusual location of patient B's tumor (skull base) led to a difficult resection and a higher risk of recurrence. Similarly, patient D had chest wall primary disease with lymphovascular invasion, which increases relapse potential. Further research is necessary on the host immune response to sarcoma and its ability to keep relapse at bay in high risk patients. We plan to continue following these patients for indications of relapse.

The false negatives and positives identified above present limitations in the usage of this diagnostic approach for detection of minimal residual disease (MRD). The primary aim of this study was to describe a method by which to identify mutations within a multi-gene panel irrespective of clinical data. However, these results remain critical considerations regarding investigations going forward. It is our hope that future studies will elucidate methods to identify mutations without the reference tumor DNA and thus limit false negative results in patients with recurrent disease. Additionally, data extrapolated from a more longitudinal study, with increased cohort size and temporal standardization of blood draws, will likely elucidate the significance of false positives in regards to MRD detection. Although this method in its nascent stages has limitations in detecting MRD, the method can be effectively utilized in order to identify genetic mutational targets for directed treatment as new modalities are developed.

Studies have shown that mutations in TP53 can be found in a large percentage of osteosarcoma samples [21]. Lorenz *et al.* identified recurrent genetic rearrangements in osteosarcoma, most frequently involving TP53, which created a deficiency of the radiation-induced DNA damage response [27]. Translocations in the first intron of TP53 have been of particular interest and were reported as an important mechanism of TP53 inactivation in Li-Fraumeni syndrome [28]. Chromosomal instability is unique to cancer cells and may contribute to the progression at multiple stages of tumor evolution [29]. Using NGS targeted sequencing, we found a translocation in intron 1 of TP53 in the plasma of a patient with clinical relapse that matched tumor material (Pt E). Interestingly, when Pt E's plasma sample was analyzed independently, without the matched tumor material, the Delly program detected evidence of the TP53 intron 1 translocation. This indicates the possibility of developing an assay that does not require primary tumor material. We tested this hypothesis further by running each plasma sample through Delly independently. An additional translocation in TP53-intron 1 was discovered in two plasma samples from patient H from whom we never had a matched primary tumor sample. Accordingly, in the future primary tumor may not be needed with clinical or molecular informative change.

Garcia-Murillas *et al.* found that ctDNA levels in plasma can be used predict relapse with high accuracy in patients in remission from metastatic breast cancer [30]. A notable finding in patients E–G is our detection of molecular biomarkers of disease prior to radiologic detection. In Patient E, we found low levels of TP53 translocation in ctDNA at week 12 of treatment, before metastases were clinically confirmed (Figure 2A). In patient F, two SNVs were found before clinical progression (Figure 2B). The varying levels of each SNV in patient F may indicate an effect on tumor heterogeneity as the patient received therapy. Finally, we discovered a ctDNA variant in plasma samples drawn from patient G both before and after radiologic progression (Figure 2C). Of note, this patient had one middle read of zero. Here too, there may be variability in the ability to collect nucleic acids relative to therapy administration time points. For this reason, standardized collection points relative to chemotherapy administration may be beneficial in future studies.

Our study was limited by a small sample size, a small number of plasma collections, and varying clinical time courses between patients. Based on the IRB protocol for minimal risk, study sample were collected during routine blood draws at office visits and on procedures days. It is our hope that expansion of the study will allow for more temporal standardization of sample collections and thus less variability in ctDNA detection relative to clinical timepoints.

A majority of the sequencing and data analysis was performed by technicians blinded to clinical outcomes. However, we were limited by a lack of blinding in the final clinical analysis. Despite these limitations, we have managed to validate our NGS method for identifying ctDNA with the mutations discovered. Though our sample size was too small to understand prognostic indications of ctDNA findings, we were encouraged by the findings in each patient with relapse.

In future studies, we plan to increase sample sizes and standardize plasma collection time points. We will to continue following the patients currently in cohort, sequencing their cell free DNA, and correlating findings with clinical outcomes. NGS has a random error rate between 0.1% and 1% [31], which limits the ability to detect ultra low frequency mutations. Therefore sanger sequencing will be used to identify lower frequency mutations and ddPCR to enhance and confirm. Additionally, increasing the number of frequently mutated genes on our panel will allow for detection of more genetic aberrations that can be followed over time. Several studies have explored osteosarcoma genotype-specific responsiveness to drugs, with Perry *et al.* identifying the PI3K/mTOR pathway as a central vulnerability for therapeutic exploitation [32]. Our future goal is to identify ctDNA in the bloodstream that can be used to predict sensitivity to drugs and direct treatment with targeted therapies.

In conclusion, our study has shown that a targeted NGS approach can be utilized to identify somatic mutations by comparing tumor and germline DNA sequences. We also demonstrated that these somatic mutations could be identified in cell free DNA isolated from serially collected plasma samples. Our future goals are to continue to validate our non-invasive method and utilize ctDNA to monitor clinical outcomes as well as investigate actionable targets identifiable by ctDNA analysis.

## MATERIALS AND METHODS

### Ethics statement

This study was approved by the Institutional Review Board. 10 patients with osteosarcoma were recruited and patients signed informed consent for use of their tissue biopsies and blood plasma samples.

### DNA extraction

Tumor biopsies were obtained from 10 patients with osteosarcoma. Each patient's tumor was biopsied either during surgery or prior to surgical removal. The biopsy specimens were converted to formalin-fixed, paraffin embedded (FFPE) tissue samples. Each scroll was 10 microns thick. DNA was extracted using GeneRead DNA FFPE kit from Qiagen. The DNA obtained from the tissue samples was quantified using nanodrop and Qubit readings.

Based on the IRB protocol for this study, patient were exposed to no more than minimal risk. Blood was drawn from patients when they presented to their primary oncologist for follow up visits and were scheduled for blood draws for clinical diagnostic purposes. Based on this, there was no standardization of sample collection relative to imaging or clinical visit timeline. Blood samples of patients were obtained and peripheral mononuclear blood cells were isolated. Germline DNA was extracted using DNeasy Blood and Tissue kit from Qiagen.

Serial blood samples were obtained from 10 patients with osteosarcoma who were at various stages of treatment or post treatment. The blood collected in Cell-Free DNA blood collection tubes. Cell free DNA was isolated from blood using the QIamp Circulating Nucleic Acid kit from Qiagen.

### Library prep, custom capture, and sequencing

Library preparations were pooled and captured using oligonucleotides for introns and exons of the 7 genes (NimblegenSeqCap) ) (Supplementary Table 4). Paired-end, 100 base Illumina sequencing runs were demultiplexed and fastq files generated with Picard Tools ExtractIlluminaBarcodes and IlluminaBclToFastq, respectively. Flanking adapter sequences were removed with Trim Galore and read quality was assessed with FastQC. Fastq files were aligned to human genome hg19 using BWA MEM, after which alignment files were sorted by coordinates and duplicates either marked or removed using Picard Tools SortSam and MarkDuplicates, respectively. Any library sequenced twice had their alignment files merged with Picard Tool MergeSamFiles, followed by re-sorting and re-marking or removing of duplicates. Depth of coverage within the regions of the 7 targeted genes was determined for duplicates-removed alignment files using with the Genome Analysis Toolkit module Depth of coverage (Supplementary Table 1) [33]. All other downstream analyses were performed with duplicates-marked alignment files.

Somatic SNVs, indels, and SVs were discovered using many recommendations suggested in the Genomic Data Commons (GDC) DNA-Seq Analysis Pipeline. Local indel realignment was performed with GATK modules Realigner Target Creator and Indel Realigner. For somatic SNV, indel, and SV discovery, local indel realignment was performed using patient-specific tumor and germline pairs of bam files as input; by contrast, for purposes of determining variant allele frequencies for a list of alleles, indel realignment was performed on individual bam files. Base quality score recalibration was subsequently performed using GATK modules BaseRecalibrator and PrintReads. For each patient-specific tumor and germline pair, somatic SNVs and indels were discovered using MuTect2 (a component of the GATK), VarScan 2 [34] and Lancet [35]. Somatic calls that passed all MuTect2 and Lancet filters were considered significant and used by downstream applications. SNVs discovered by VarScan 2 somatic were filtered further with VarScan 2 module processSomatic, followed by fpfilter, as described in Koboldt *et al.* [36]. and only these high-confidence calls were used by downstream applications. Indels identified by VarScan 2 were excluded from further analysis, as suggested by the GDC DNA-Seq Analysis Pipeline. VCF files containing each patient's high-confidence somatic mutations discovered by the three somatic callers were compressed, then combined using bcftools concat (which also removes duplicates) and sorted with vcftools vcf-sort to generate a single master list of somatic variants per patient. Each master list was used as input to GATK HaplotypeCaller to obtain allele frequencies for patient-specific tumor, germline, and cfDNA samples. Somatic mutations were excluded from further analysis if the tumor sample's variant allele frequency was less than 0.05. To identify potentially pathogenic somatic variants, HaplotypeCaller-generated VCF files were annotated using SnpEff [37].

Somatic SVs were discovered with DELLY2 using modules call and filter and patient-specific tumor and germline bam files as input [38]. SVs supported by sufficient reads to permit precise identification of breakpoints and passing all DELLY filters were considered significant. High-confidence somatic SVs discovered in this manner were genotyped and their reference and variant read counts were determined for patient-specific tumor, germline, and cfDNA samples using DELLY module call. Lastly, to explore the possibility of identifying somatic translocations in the absence of discovery using patient-specific tumor and germline sample pairs, all tumor, germline, and cfDNA samples were run individually through the DELLY2 module call; output vcf files were filtered, using ad-hoc scripts, to identify any SV calls that passed all filters, supported sufficient reads to permit precise identification of a breakpoint, and the first or second chromosomal position of the call was located within intron 1 of the *TP53* gene, a common site of translocations in osteosarcoma (Supplementary Table 5).

## SUPPLEMENTARY MATERIALS TABLES



## Abbreviations

cfDNA: cell free DNA
tDNA: circulating tumor DNA
gDNA: genomic DNA
INDEL: insertions and deletions
MAP: therapy: Methotrexate, Doxorubicin & Cisplatin
MRD: minimal residual disease
NED: no evidence of disease
SNV: single nucleotide variant
SV: structural variant
