# Supplementary material for: Detection of circulating tumor DNA in patients with osteosarcoma
Source: Oncotarget. 2018 Jan 18;9(16):12695–704. doi: 10.18632/oncotarget.24268 (PMC5849166; doi:10.18632/oncotarget.24268)
Supplement: Supplementary file 1 [file oncotarget-09-12695-s001.pdf]

Detection of circulating tumor DNA in patients with osteosarcoma

SUPPLEMENTARY MATERIALS

Supplementary Table 1: Mean coverage of NGS in patient samples

Type	Mean	Median	Range
All Samples	698.4.1x	635.8x	69–1,524
Tissue Biopsy	514.1x	565.2x	69–763
gDNA	1,343.5x	1363x	1167–1524
ctDNA	557.3x	550.2x	133.3–1009.2

Supplementary Table 2: Variants discovered in ctDNA

				Reads (Reference:Variant)		
Patient	Mutation type	Gene	Chr:Position	Tumor	gDNA	ctDNA by sample
B	SNV	*DLG2*	11:83342241	293:35	1070:1	596:11, 442:17, 349:30, 262:22, 640:0
D	SNV	*DLG2*	11:84804137	305:30	1037:0	547:36, 749:0, 552:66, 153:0
E	Translocation	*TP53* intron 1	17:7583675 with 6:37227977	221:67	675:0	243:0, 128:1, 180:239, 331:3
F	SNV	*ATRX*	X:77033528	94:81	365:0	109:4, 183:0, 172:0, 95:0
F	SNV	*DLG2*	11:84514691	377:208	2193:1	621:5, 866:1, 1059:0, 1137:0
G	SNV	*MET*	7:116427288	521:55	1453:1	721:52, 722:0, 1104:70, 187:0
H	Translocation	TP53 intron 1	17:7588196 with 22:43579550	N/A	364:0	186:0, 246:38, 54:15

Supplementary Table 3: Pathogenic variants discovered in tumor samples

a. SNVs

Patient	Gene	rs #	Mutation	Tumor (Reference:Variant)	gDNA (Reference:Variant)
A	*TP53*	rs28934578	p.Arg175His	17	0.002
B	*TP53*	rs28934874	p.Pro151Ser	38	0
D	*RB1*	rs1131690907	Splice donor site	32	0.07

b. SVs

Patient	Mutation type	Gene	Chr:Position	Tumor (Reference:Variant)	gDNA (Refernce:Variant)
E	Translocation	*TP53* intron1	17:7583675 with 6:37227977	221:67	675:0
G	*Deletion*	*ATRX*	X:76894261 intron 2-15	154:25	382:0

Supplementary Table 4: Custom designed oligonucleotide probes

Gene	Chromosome	Size (bases)	Bases-Probe Coverage	Fraction Probe Coverage
MET	chr7	125997	100852	0.8
PTEN	chr10	108818	84808	0.779
DLG2	chr11	2172912	1733709	0.798
RB1	chr13	178236	125303	0.703
TP53	chr17	25760	13338	0.518
SLC19A1	chr21	50840	45271	0.89
ATRX	chrX	281347	175162	0.623

Supplementary Table 5: Bioinformatics tools

Tool	Version	Link
Picard Tools	1.119 and 2.3.0	http://broadinstitute.github.io/picard/
Trim Galore	0.3.7	https://www.bioinformatics.babraham.ac.uk/projects/trim_galore/
FastQC	0.11.4	https://www.bioinformatics.babraham.ac.uk/projects/fastqc/
BWA	0.7.10	http://bio-bwa.sourceforge.net/bwa.shtml
Genome Analysis Toolkit (includes Mutect2 & HaplotypeCaller)	3.5	https://software.broadinstitute.org/gatk/
Genomic Data Commons DNA-Seq Analysis Pipeline		https://docs.gdc.cancer.gov/Data/Bioinformatics_Pipelines/DNA_Seq_Variant_Calling_Pipeline/
fpfilter		https://github.com/ucscCancer/fpfilter-tool
bcftools	1.3.1	http://www.htslib.org/doc/bcftools.html
vcftools	0.1.12b	https://vcftools.github.io/man_latest.html
DELLY2	0.7.5	https://github.com/dellytools/delly
SnpEff	4.2	http://snpeff.sourceforge.net
VarScan 2	2.4.3	http://dkoboldt.github.io/varscan/
Lancet	1.0.0	https://github.com/nygenome/lancet
